# Multilevel landscape utilization of the Siberian flying squirrel: Scale effects on species habitat use

**DOI:** 10.1002/ece3.3359

**Published:** 2017-09-07

**Authors:** Jaanus Remm, Ilpo K. Hanski, Sakari Tuominen, Vesa Selonen

**Affiliations:** ^1^ Department of Biology Section of Ecology University of Turku Turku Finland; ^2^ Department of Zoology Institute of Ecology and Earth Sciences University of Tartu Tartu Estonia; ^3^ Kirkkotie 127A Siuntio Finland; ^4^ Natural Resources Institute Finland Helsinki Finland

**Keywords:** distribution modeling, metapopulation, pattern decomposition, population configuration, protection planning, scale dependency

## Abstract

Animals use and select habitat at multiple hierarchical levels and at different spatial scales within each level. Still, there is little knowledge on the scale effects at different spatial levels of species occupancy patterns. The objective of this study was to examine nonlinear effects and optimal‐scale landscape characteristics that affect occupancy of the Siberian flying squirrel, *Pteromys volans*, in South‐ and Mid‐Finland. We used presence–absence data (*n* = 10,032 plots of 9 ha) and novel approach to separate the effects on site‐, landscape‐, and regional‐level occupancy patterns. Our main results were: landscape variables predicted the placement of population patches at least twice as well as they predicted the occupancy of particular sites; the clear optimal value of preferred habitat cover for species landscape‐level abundance is a surprisingly low value (10% within a 4 km buffer); landscape metrics exert different effects on species occupancy and abundance in high versus low population density regions of our study area. We conclude that knowledge of regional variation in landscape utilization will be essential for successful conservation of the species. The results also support the view that large‐scale landscape variables have high predictive power in explaining species abundance. Our study demonstrates the complex response of species occurrence at different levels of population configuration on landscape structure. The study also highlights the need for data in large spatial scale to increase the precision of biodiversity mapping and prediction of future trends.

## 1 INTRODUCTION

Animals use and select habitat at multiple hierarchical levels and at different spatial scales within each level (McGarigal et al., 2016; Wiens, 1989). At the local level, site occupancy is determined by local habitat and community characteristics (Dunning et al., 1992; Van Buskirk, 2005). At the broader level, processes such as movement of individuals between local populations, regional patterns in landscape structure, or species interactions also affect occupancy patterns (Boscolo & Metzger, 2011; Dunning et al., 1992). Consequently, the spatial scale at which habitats affect the species differ at different levels that result from individuals behaviors in space, such as home ranges, population patches, or ranges (McGarigal et al., 2016).

The hierarchical nature and scale dependency of habitat use complicates evaluation of the habitat characteristics required to support populations. For example, the commonly used approach to determine conservation or management units from the habitat requirements of individuals may not be appropriate tool for securing population persistence (Jackson & Fahrig, 2015). Rather, knowledge of the spatial distribution of habitats, populations, population subunits, and individuals is a key element for the success of conservation and management programs. However, there is currently little understanding of how processes at different scales simultaneously influence species occupancy patterns (Du Toit, 2010; McGarigal et al., 2016; Wheatley et al., 2005).

Arboreal mammals are an animal group that have very explicit habitat and dispersal limitations and thus are interesting species to study scale‐dependent habitat utilization. They depend on forest habitat, which is often heavily managed. Many of these species are reluctant to cross large forest gaps (Bakker & Van Vuren, 2004; van der Ree et al., 2010) and may, therefore, be unable to colonize suitable and empty habitat patches (but see Selonen & Hanski, 2004; Fey et al., 2016). Consequently, the distribution and abundance patterns of arboreal mammals are frequently influenced by habitat composition and configuration at both the patch and landscape level (Hurme et al., 2008; Mortelliti et al., 2011; Nupp & Swihart, 2000; Pardini et al., 2005). One such species is the Siberian flying squirrel (*Pteromys volans*), a forest‐dwelling rodent with a declining population trend (Hanski, 2006; Jokinen et al., 2015; Santangeli et al., 2013a; Selonen et al., 2010). Habitat selection of the flying squirrel is well studied at the site level (i.e., selection of nesting and feeding sites; Hanski, 1998; Reunanen et al., 2002), but also at the landscape level (Hurme et al., 2008; Reunanen et al., 2000; Santangeli et al., 2013b). However, as is the case for other arboreal rodents, we lack knowledge about scale‐dependent landscape effects on population configuration. The species requires nest cavities that are usually found in old aspens (*Populus tremula*) in Norway spruce (*Picea abies*)‐dominated forests (Hanski, 1998; Reunanen et al., 2002). The distribution of aspen and spruce is in turn correlated with the presence of fertile soils, which are frequently used for agricultural cultivation. Thus, and somewhat surprisingly, an earlier study found a positive correlation between flying squirrel occurrence and the presence of fields in the landscape (Santangeli et al., 2013b). In the long term, however, agricultural land use creates large areas of unsuitable open habitat in landscapes, which, depending on the dispersal potential of the species, negatively affects the population distribution of arboreal species (Van Apeldoorn et al., 1994). In other words, the response of flying squirrels to certain landscape variables, such as agricultural areas, should be nonlinear.

The objective of this study was to examine scale dependency and nonlinear effects of the landscape characteristics that affect occupancy patterns of flying squirrels. We expect habitat variables to affect at different spatial scales, when studied at different levels of population configuration (compare to multilevel and multiscale definition by McGarigal et al., 2016). In our case, the lowest level is (1) the occupancy of study site (hereafter site occupancy). At this level, we expect landscape variables to dominate at spatial scales that describe the home‐range utilization of individuals. At the broader level, (2) the abundance of occupied sites in the landscape (hereafter landscape‐level abundance) may also be related to processes that are affected by landscape variables that operate at larger spatial scale than home range (like movement of individuals). We define the landscape‐level abundance to depend on the configuration of functional subpopulation patches. Subpopulation patches are the areas where a species' local average abundance is higher than in surroundings. Finally, we predict that large‐level (3) regional patterns in population density within the studied distribution range (hereafter regional density) may affect landscape utilization at site, and landscape levels (levels 1 and 2 above), that is, the responses may vary in areas of high and low population density (see e.g., Mazerolle & Villard, 1999; Sundell et al., 2012).

## 2 METHODS

### 2.1 Study species and study area

**Figure 1:**
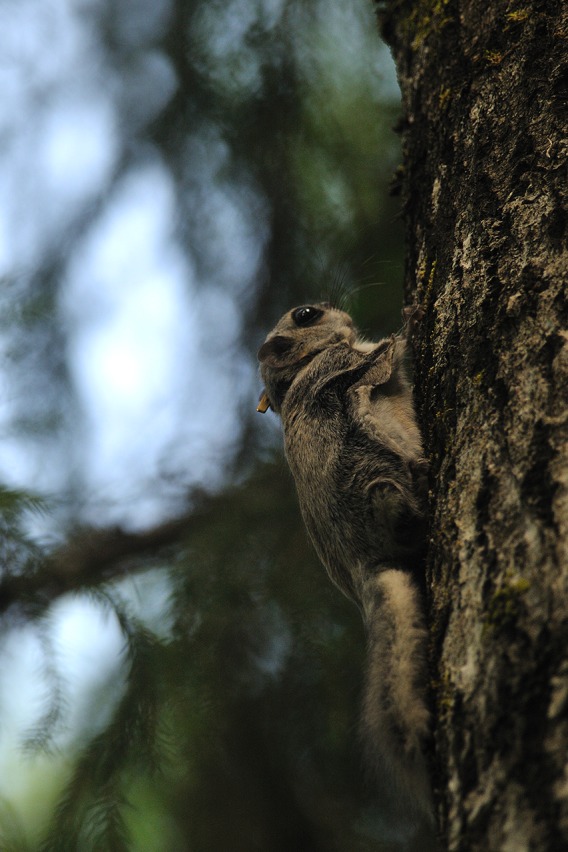
The study organism, Siberian flying squirrel. Photo: M. Absalon

The flying squirrel is a small arboreal rodent (Fig. 1) distributed from eastern Siberia and Japan to Europe, where the species is found only in Finland, Estonia, and Russia (Ognev, 1966; Wilson & Reeder, 2005). In Finland, flying squirrels occur from southern Lapland to the southernmost coast of the country. The species is mainly associated with mature and old spruce‐dominated mixed forests containing deciduous trees for foraging and with cavities for nesting and roosting (Hanski et al., 2000a; Santangeli et al., 2013b). Adults are site‐tenacious, with home ranges averaging 8 ha for females and 60 ha for males (Hanski et al., 2000a; Selonen & Wistbacka, 2017). Mean natal dispersal distances are between 1 and 2 km, with a maximum of almost 9 km (Hanski & Selonen, 2009).

The study area included South‐ and Mid‐Finland between 59.96°N, 21.20°E and 65.87°N, 31.50°E, covering 177,630 km². The area covers the majority of potential habitat for flying squirrels in Finland. Forests dominate the Finnish landscape, with the most common tree species being Scots pine (*Pinus sylvestris*) and Norway spruce, with a lower proportion of aspen, birch (*Betula* spp.), and other deciduous trees (such as grey alder, *Alnus incana*, and black alder, *A. glutinosa*). Most forests in Finland are managed for timber production. Other important landscape types are agricultural fields, lakes, and peatlands.

### 2.2 Data collection

We used presence–absence data for flying squirrels obtained from 9 ha study site (300 × 300 m; *n* = 10,032) surveyed in 2003–2005. The survey was originally organized by Finnish Ministry of Environment (Hanski, 2006) to get knowledge on population status of the flying squirrel in Finland, because the species is protected by EU habitats directive. Size of the study site was selected to match average female home range (8.3 ha, Hanski et al., 2000b). The study sites were placed randomly in forest habitats with an average density of 10 sites per 200 km² (10 plots in every second 10 × 10 km square; for more information, see Santangeli et al., 2013b). The sites were located on mineral soil and were at least 1 km apart. They were inspected for signs of flying squirrel activity once by searching extensively for the presence of fecal droppings. This is a commonly adopted protocol for surveying flying squirrels (e.g., Hurme et al., 2005, 2008; Mönkkönen et al., 1997; Reunanen et al., 2002). All surveys were carried out between April and June by trained surveyors. Pellets are relatively easy to find due to their yellow color and deposition location: usually at the bottom of large aspen and spruce trees. Therefore, detection probably can be assumed to be close to one (Santangeli et al., 2013b) and the risk of false absences to be low in our data (Hurme et al., 2008). Thus, we have no reason to expect detection efficiency to be uneven within the surveyed area and do not anticipate spatial bias in the results.

### 2.3 Analysis

**Figure 2:**
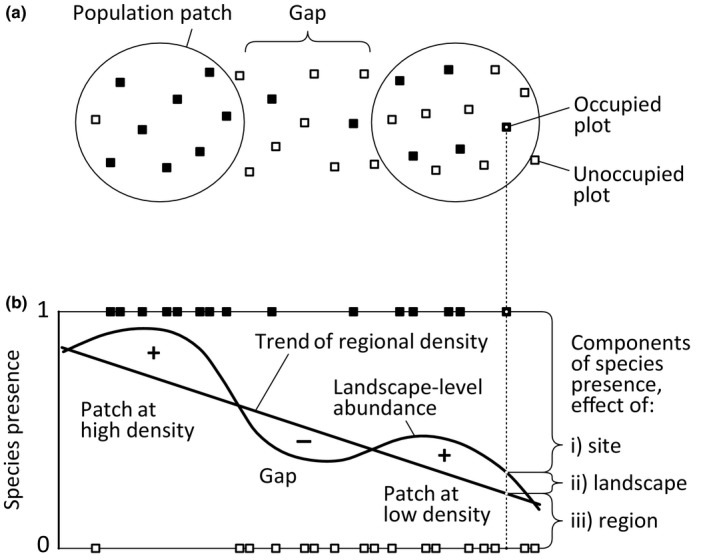
Principle of decomposing species presence–absence information: (a) population spatial pattern and sampling (arrangement simplified on the chart); (b) three additive components of species presence (i, ii, iii) projected on the diagram using one dimension of the study area. Note that two‐dimensional models were used on the real data. Population patches and gaps are defined as areas of higher (+) and lower (−) local abundance in relation to the global trend of regional density. In the analysis, gross values of site occupancy (i + ii + iii) and landscape‐level abundance (ii + iii) were explained by landscape metrics, while the partial components of landscape‐level abundance (ii) and regional density (iii) were used as covariates accounting for the effects of lower level patterns

We distinguished three levels in the information of species presence–absence: site occupancy—present or absent (0 or 1), landscape‐level abundance—proportion of occupied sites (0–1) within the range of spatial dependence (i.e., autocorrelation), and regional density—global spatial trend of occupancy proportion (0–1; Fig. 2). We build two models: In the first model response variable was the site occupancy. In the second model, response variable was the landscape‐level abundance. The study site was, however, the unit of analysis in both models, because for landscape‐level abundance, we used site‐specific values based on components of universal kriging (local weighted mean). When analyzing site occupancy, we included landscape‐level abundance and regional density as covariates in the model alongside other landscape variables. For the landscape‐level abundance model, regional density was included as a covariate. Regional density based on trend surface component of the universal kriging. It was not modeled separately, but was used to analyze interactive effects in site occupancy and landscape‐level abundance models.

The step‐by‐step details of the data management and statistical analysis are explained subsequently.

#### 2.3.1 Estimation of regional density and landscape‐level abundance with the geostatistical method universal kriging

That accounts spatial dependence of a variable as two components: large‐scale trend surface interpolation (regional effects), and local structures of spatial autocorrelation (landscape patchiness) (Lam, 1983; Legendre & Fortin, 1989). The two‐scale procedure is about 40% more effective for describing spatial patterns than ordinary kriging, which does not consider the large‐scale trend (Ghiasi & Nafisi, 2015).

First, we estimated a two‐dimensional quadratic trend surface model to explain species distribution density as a global gradient (two‐way binomial regression). This was the regional density, that is, the largest level (level 3 in introduction), we used to describe the population configuration of flying squirrels. Second, using the residual values from the trend surface, a spherical semivariogram was modeled to describe the spatially autocorrelated portion of the population density structure in landscape level (Fig. S1). The subsequent weights of spatial dependence between sampling sites were based on the semivariogram model. Third, we estimated distance‐weighted average occupancy of surrounding sites within the semivariogram range for each site. This was the landscape‐level abundance of the species, that is the medium level (level 2 in introduction), used to describe population configuration of the flying squirrel. In other words, the landscape‐level abundance described average occupancy rate at the scale of population patches estimated by the kriging method.

#### 2.3.2 Selection of explanatory landscape variables

We used five variables that are known or expected to correlate well with habitat and landscape utilization of flying squirrels.

1. Mature spruce‐deciduous forest (estimated > 50 years old; hereafter preferred habitat), which earlier studies indicate to be the preferred habitat of flying squirrels (Hanski, 1998; Reunanen et al., 2002; Santangeli et al., 2013b; Selonen et al., 2001). We measured the proportion of the preferred habitat within six radii around the centers of study sites: 100 m, 250 m, 500 m, 1 km, 2 km, and 4 km. Among the radii, the most parsimonious one was used in the final models, selected in the third step of the analysis (2.3.3; same for the rest of the variables). Habitat data from the same year as sampling of flying squirrels were based on the multisource national forest inventory (MS‐NFI) in Finland (www.paikkatietoikkuna.fi), which employs satellite images, digital maps, and field measurements for producing forest estimates in the form of thematic maps (Tomppo et al., 2008). The thematic maps include, among others, volumes of main tree species: pine, spruce, birches, and other broadleaved trees.
2. Aspen and alders, key tree species related to nest site presence and foraging conditions (Hanski et al., 2000a; Selonen & Wistbacka, 2016). Data for these trees are not presented in MS‐NFI maps, but we estimated the growing stock volume (m³/ha) of these species within a 1 km² that contains each study site by combining NFI field measurement plots and MS‐NFI thematic forest maps. Based on volumes of aspen, grey alder, and black alder calculated for each NFI field plot (Tomppo et al., 2008), we created thematic map layers presenting the volumes of aspen, grey alder, and black alder. These map layers were produced by dividing the pixel values of the map layer “volume of broadleaved trees” into strata representing aspen, grey alder, and black alder, based on geostatistical interpolation of the volume proportions of the respective tree species in NFI plots. Inverse distance weighting of the geographically nearest 12 NFI field plots was used in the geostatistical interpolation. The outcome of the interpolation was volume maps with similar resolution as the original MS‐NFI thematic maps. Based on these maps, the volumes of the three tree species were then calculated for 1 km² grid elements, forming a uniform map grid covering the entire study region.
3. Soil fertility (rank of 1, high to 8, low fertility), representing the long‐term suitability of land for deciduous tree species and spruce, as well as for agriculture. We measured the average site fertility index at six radii around the centers of study sites: 100 m, 250 m, 500 m, 1 km, 2 km, and 4 km. The fertility index based on ranks using habitat type as a proxy for fertility: 1. Herb‐rich sites, 2. Herb‐rich heath forests, 3. Mesic, 4. Subxeric forests, 5. Xeric forests, 6. Barren forests, 7. Rocky and sandy soils, and 8. Summit and fjeld forest. This is commonly used proxy to describe soil fertility in Finland (Tomppo et al., 2008).
4. Forest cover represents landscape connectivity for the species that prefer to not cross open habitats. We measured the proportion of the area covered by forest at eight radii around centers of study sites: 100 m, 250 m, 500 m, 1 km, 2 km, 4 km, 10 km, and 20 km. Forest cover within radii of 10 km and 20 km was measured from CORINE land cover map data (European Environment Agency; proportion measurements for other radii were taken from MS‐NFI data), as they might reflect very large‐scale effects on the dispersal ability of the species, although these radii were not available for landscape variables (i), (ii), and (iii).
5. Agricultural fields represent an expected negative coeffect at otherwise favorable fertile soils. We measured the proportion of fields at eight radii around centers of study sites: 100 m, 250 m, 500 m, 1 km, 2 km, 4 km, 10 km, and 20 km. Agricultural field cover within the 10 km and 20 km radii was calculated from CORINE land cover map data.

#### 2.3.3 Univariate models for every landscape variable for site occupancy and landscape‐level abundance

We used nonparametric, nonlinear generalized additive models (GAM; Hastie & Tibshirani, 1986; Li & Wang, 2013). Among several statistical methods used for studying species habitat selection, GAMs have a good optimum of flexibility for input data types, computational complexity, and popularity in the scientific community (Li & Wang, 2013).

We compared the effects of landscape variables at all measured radii in models for site occupancy and landscape‐level abundance. The measurements of landscape variables within different radii are intercorrelated (Table S1). Thus, for further analysis (multivariate GAM), we selected the radius at which the correlation of each landscape variable was strongest with site occupancy and the landscape‐level abundance of the species (DeCesare et al., 2012; Wheatley, 2010). Selection was based on the Akaike Information Criterion (AIC, Johnson & Omland, 2004; Anderson, 2008), which measures model parsimony. As a result, we maximized the ecologically relevant information that was entered into the final step of the analysis (multivariate GAM).

#### 2.3.4 Multivariate GAM

We used the landscape variables at the radii selected based on the univariate models described above. In the multivariate model (GAM) selection procedure, all possible combinations of the explanatory variables were compared along with their interactions with regional density of flying squirrels. The interactive terms were used to test for a difference in habitat use between sites with low and high regional density and to account geographically variable landscape effects. Selection of the best set of explanatory variables was based on the AIC.

All explanatory variables and covariates were continuous variables. The only categorical variable in the models was the response variable site occupancy, measured as presence–absence (binomial variable). As variation in the explained variables was limited between 0 and 1, a logit link‐function was used to generate normal residual distributions, and to ensure that all fitted values lay between 0–1. In the site occupancy models, variogram‐based landscape‐level abundance was used as a covariate covering the effect of spatial autocorrelation. In the landscape‐level abundance model, we incorporated spherical spatial correlation structure in the GAM to account the effect of spatial autocorrelation. To avoid over‐fitted models, the degrees of freedom (*df*) for smoothing splines was limited to *df* ≤ 2. R packages ape (Paradis et al., 2004), gstat (Pebesma, 2004), mgcv (Wood, 2011), and sp (Bivand et al., 2013; Pebesma & Bivand, 2005) were used.

## 3 RESULTS

### 3.1 Site occupancy model

**Figure 3:**
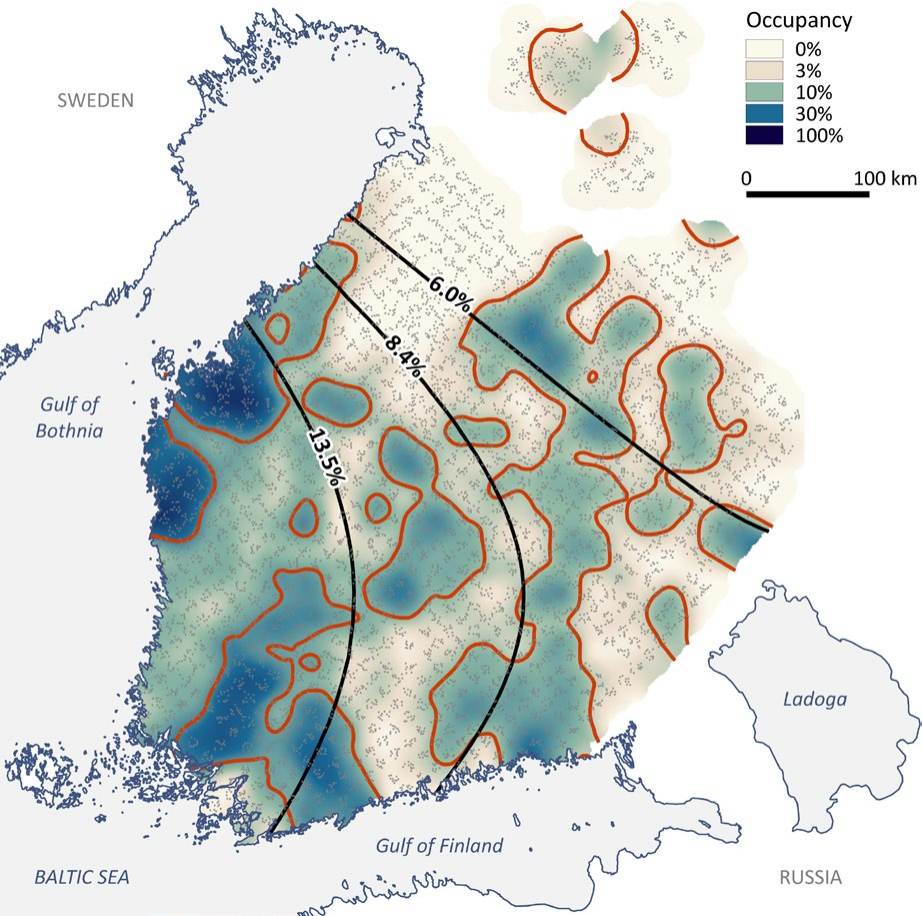
Geographic distribution of flying squirrel presence in Finland. The density distribution estimate is calculated using universal kriging. The small gray dots represent the 10,032 study sites (9 ha); black lines denote isoclines of large‐scale regional density quartiles according to the global quadratic trend surface model; the red lines represent isoclines of average population density at the landscapes scale, relative to the global trend model

In total, 10,032 plots in forest habitats were studied for flying squirrel occupancy. About 10.3% of the plots were occupied (1,030 plots, Fig. 3). According to the selection of optimal spatial scale from univariate models, the strongest correlations between site occupancy and landscape variables occurred with the proportion of preferred mature spruce‐deciduous forest habitat within the 250 m radius, followed by three metrics within the 500 m radius: proportion of forest cover, average soil fertility index, and proportion of fields (see the Δ_AIC_ 1 and 2 in the Table 1). That is, the relationships between 9 ha site occupancy and landscape variables were strongest within radii that represent a several times larger area around a study site than the plot itself (250 m radius = 19.63 ha, 500 m radius = 78.54 ha).

**Table 1:** Comparison of univariate effects of environment variables at different scales (radius around study site). Besides a landscape variable, coarser levels of flying squirrel population configuration are used as covariates in each GAM. An asterisk denotes the radius of the variable selected for further multivariate analysis; Δ_AIC_ 1 represents the particular AIC difference from the best predictor over all variables and radii; Δ_AIC_ 2 represents the AIC difference from the best radius among the same variable

Variable, within radius	Site occupancy 9 ha square			Landscape‐level abundance 32.5 km radius		
	AIC	Δ_AIC_ 1	Δ_AIC_ 2	AIC	Δ_AIC_ 1	Δ_AIC_ 2
Average soil fertility index						
*r* = 100 m	5,266.4	64.0	46.0	42,224.5	1,458.4	1,311.8
*r* = 250 m	5,222.8	20.4	2.4	41,865.8	1,099.7	953.1
*r* = 500 m	5,220.4	18.0	0.0*	41,557.4	791.3	644.7
*r* = 1 km	5,240.0	37.6	19.6	41,323.7	557.6	411.0
*r* = 2 km	5,266.8	64.4	46.4	41,108.2	342.1	195.5
*r* = 4 km	5,297.8	95.4	77.4	40,912.7	146.6	0.0*
Proportion of agricultural areas						
*r* = 100 m	5,339.4	137.0	122.6	43,318.6	2,552.5	153.9
*r* = 250 m	5,280.7	78.3	63.9	43,249.4	2,483.3	84.7
*r* = 500 m	5,216.8	14.4	0.0*	43,170.2	2,404.1	5.5
*r* = 1 km	5,248.7	46.3	31.9	43,164.7	2,398.6	0.0*
*r* = 2 km	5,290.7	88.3	73.9	43,193.1	2,427.0	28.4
*r* = 4 km	5,324.7	122.3	107.9	43,231.7	2,465.6	67.0
*r* = 10 km	5,339.0	136.6	122.2	43,304.7	2,538.6	140.0
*r* = 20 km	5,328.5	126.1	111.7	43,231.6	2,465.5	66.9
Proportion of forest cover						
*r* = 100 m	5,327.2	124.8	111.1	43,280.9	2,514.8	551.9
*r* = 250 m	5,326.2	123.8	110.1	43,166.9	2,400.8	437.9
*r* = 500 m	5,216.1	13.7	0.0*	42,919.7	2,153.6	190.7
*r* = 1 km	5,218.4	16.0	2.3	42,818.0	2,051.9	89.0
*r* = 2 km	5,251.9	49.5	35.8	42,742.9	1,976.8	13.9
*r* = 4 km	5,284.5	82.1	68.4	42,729.0	1,962.9	0.0*
*r* = 10 km	5,332.3	129.9	116.2	43,208.3	2,442.2	479.3
*r* = 20 km	5,319.6	117.2	103.5	43,252.4	2,486.3	523.4
Proportion of mature spruce and deciduous forests						
*r* = 100 m	5,250.4	48.0	48.0	42,692.7	1,926.6	1,926.6
*r* = 250 m	5,202.4	0.0	0.0*	42,240	1,473.9	1473.9
*r* = 500 m	5,252.9	50.5	50.5	41,768.4	1,002.3	1,002.3
*r* = 1 km	5,287.4	85.0	85.0	41,387.6	621.5	621.5
*r* = 2 km	5,308.8	106.4	106.4	41,031.9	265.8	265.8
*r* = 4 km	5,308.2	105.8	105.8	40,766.1	0.0	0.0*
Stock volume within 1 km²						
Aspen	5,330.9	128.5	0.0*	43,053.6	2,287.5	179.0*
Grey alder	5,334.9	132.5	4.0*	42,874.6	2,108.5	0.0*
Black alder	5,341.0	138.6	10.1*	43,213.3	2,447.2	338.7*

**Figure 4:**
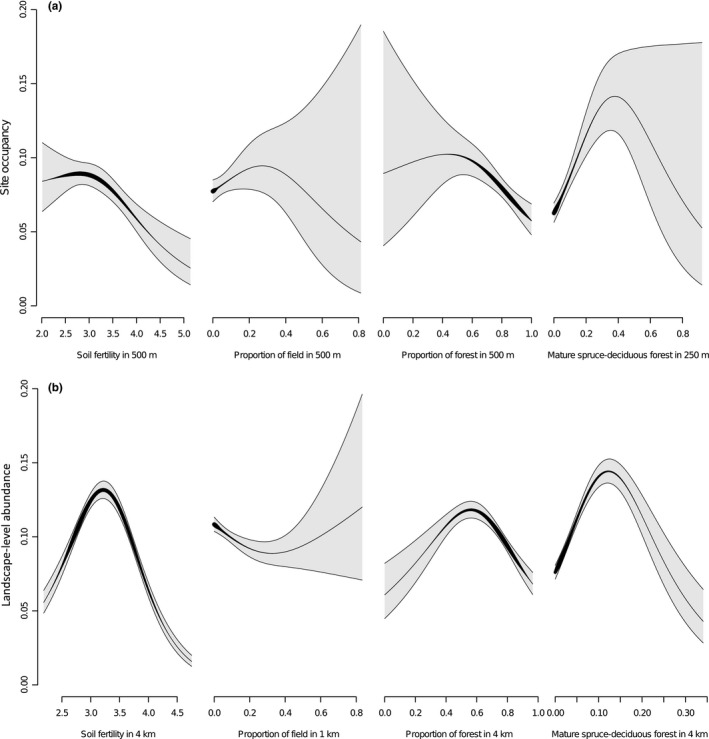
Partial effects of four landscape variables in multivariate GAM on (a) occupancy of 9 ha sites and (b) landscape‐level abundance (patches of 32.5 km radius). Gray areas represents 95% confidence intervals; line width represents sampling density. See details on model parameters in Table 3

In the multivariate model selection, two of the highest ranked candidate models gained similar explanatory power (Δ_AIC_ = 1.9, Table 2a). These two models included the four variables listed above along with stock volumes of grey and black alder, but the importance of the latter was low (Table 2a). Based on the first ranked multivariate model (*R* ^2^ = 0.21, Table 3a), average soil fertility, proportion of forest cover, and proportion of preferred habitat explained most variation in site occupancy. Most of the studied landscape features were strongly nonlinearly correlated with presence of flying squirrel (see the *df* > 1 values in the Table 2a and Fig. 4a): The effect of average soil fertility index increased toward higher fertility that is low index values; the proportion of fields had a positive effect up to 30% of landscape composition; the proportion of forest cover had a negative effect at higher than 50% of landscape composition; and a positive effect of preferred habitat was apparent up to 35% of landscape composition (Fig. 4a).

**Table 2:** Variable importance among 254 candidate models of flying squirrel site occupancy and 254 candidate models of landscape‐level abundance, and variable representation in the five models of highest AIC weight. Δ_AIC_ values are relative to the best site occupancy model AIC = 5,049.6, and the best landscape‐level abundance model AIC = 40,235.3. + indicates that a variable was present in the model

Rank of the model	Importance	5 Best‐fitted models				
		1.	2.	3.	4.	5.
(a) Site occupancy						
Δ_AIC_		0.0	1.9	2.6	4.4	5.0
AIC weight		0.497	0.196	0.134	0.054	0.042
Average soil fertility index in 500 m radius	0.998	+	+	+	+	+
Proportion of fields in 500 m radius	0.997	+	+	+	+	+
Proportion of forest in 500 m radius	0.999	+	+	+	+	+
Proportion mature spruce‐deciduous stands in 250 m radius	1.000	+	+	+	+	+
Stock of aspen within 1 km²	0.228			+	+	
Stock of grey alder within 1 km²	0.909	+	+	+	+	
Stock of black alder within 1 km²	0.280		+		+	
(b) Landscape‐level abundance						
Δ_AIC_		0.0	2.0	9.4	11.8	12.0
AIC weight		0.727	0.262	0.006	0.002	0.002
Average soil fertility index in 4 km range	1.000	+	+	+	+	+
Proportion of fields in 1 km range	0.997	+	+	+		+
Proportion of forest in 4 km range	0.999	+	+	+	+	+
Proportion of mature spruce‐deciduous stands in 4 km range	1.000	+	+	+	+	+
Stock of aspen within 1 km²	0.992	+	+		+	
Stock of grey alder within 1 km²	0.264		+			+
Stock of black alder within 1 km²	0.999	+	+	+	+	+

**Table 3:** The best multivariate models (*n* = 10,032) of (a) site occupancy (model: *df* = 10,015.71, *R* ^2^ = 0.21) and (b) landscape‐level abundance of flying squirrels (model: *df* = 10,013.35, *R* ^2^ = 0.44). See Fig. 4 for visual presentation of the modeled effects. The variable *df* values reflect whether an effect is linear (*df* = 1.0) or nonlinear (*df* > 1.0). To avoid over fitting of the models, limit was set at *df* ≤ 2

	Main effect			Interaction with regional density		
	*df*	Statistic	*p*	β ± *SE*	Statistic	*p*
(a) Site occupancy, in 9 ha square						
Average soil fertility index in 500 m radius	1.9	χ² = 10.31	<.001	5.91 ± 1.95	*z* = 3.03	.002
Proportion of fields in 500 m radius	1.6	χ² = 1.94	.272	17.01 ± 7.58	*z* = 2.25	.025
Proportion of forest in 500 m radius	1.8	χ² = 27.20	<.001	11.10 ± 6.03	*z* = 1.84	.066
Proportion mature spruce‐deciduous stands in 250 m radius	1.9	χ² = 32.30	<.001	−1.76 ± 5.66	*z* = −0.31	.756
Stock of grey alder within 1 km²	1.0	χ² = 8.16	.004	0.41 ± 0.17	*z* = 2.44	.014
Species landscape‐level abundance	1.0	*z* = 25.42	<.001			
Species regional density	1.0	*z* = −2.75	.006			
(b) Landscape‐level abundance, in 32.5 km radius						
Average soil fertility index in 4 km radius	2.0	*F* = 175.40	<.001	8.11 ± 1.18	*t* = 6.88	<.001
Proportion of fields in 1 km radius	1.8	*F* = 12.59	.001	6.45 ± 2.96	*t* = 2.18	.029
Proportion of forest in 2 km radius	1.9	*F* = 24.28	<.001	5.25 ± 2.84	*t* = 1.85	.064
Proportion of mature spruce‐deciduous stands in 4 km radius	2.0	*F* = 321.15	<.001	−160.06 ± 9.89	*t* = −16.18	<.001
Stock of aspen within 1 km²	1.9	*F* = 12.57	<.001	0.20 ± 0.06	*t* = 3.15	.002
Stock of black alder within 1 km²	1.0	*F* = 16.91	<.001	−0.77 ± 0.14	*t* = −5.49	<.001
Species regional density	1.0	*t* = −1.62	.106			

### 3.2 Landscape‐level abundance model

The semivariogram analysis revealed evident patchy landscape‐level abundance of flying squirrel occurrence (*R* ^2^ = 0.17; Fig. 3). The average size of patchy population structures was approximately 32.5 km (semivariogram range), and according to the spherical semivariogram model, spatial autocorrelation of neighboring sites was halved at a distance of 11.3 km (Fig. S1).

Comparison of univariate models of different spatial scales showed that landscape‐level abundance was most strongly correlated with the proportion of preferred habitat, followed by soil fertility (see the Δ_AIC_ 1 and 2 in Table 1). Both of these variables correlated most strongly with landscape‐level abundance at the largest radii studied (4 km radius). This suggests that the true optima probably lie beyond the range of studied scales. The next most important variables were forest cover within the 4 km radius and proportion of fields within the 1 km radius (Table 1).

The best multivariate model for landscape‐level abundance (*R* ^2^ = 0.44, Tables 2b and 3b) included all variables except grey alder, which was included in the second highest ranked model (full model; Table 2b). While the importance of other variables over all candidate models was high, grey alder had relatively low importance (Table 2b). In particular, the proportion of preferred habitat and the average soil fertility index had strong effects on landscape‐level abundance (Table 3b). The effects of the four most important landscape metrics according to the best multivariate model are summarized as follows (Fig. 4b): Average soil fertility index had a sharp optimum between average rank of 3.0–3.5; the proportion of forest cover showed an optimum between 50%–60% landscape composition; the proportion of agricultural fields showed a slight negative effect below 30% of landscape composition; and the proportion of preferred mature spruce‐deciduous stands showed a relatively narrow optimum between 10%–15% of landscape composition.

### 3.3 Differences at high and low regional density

**Figure 5:**
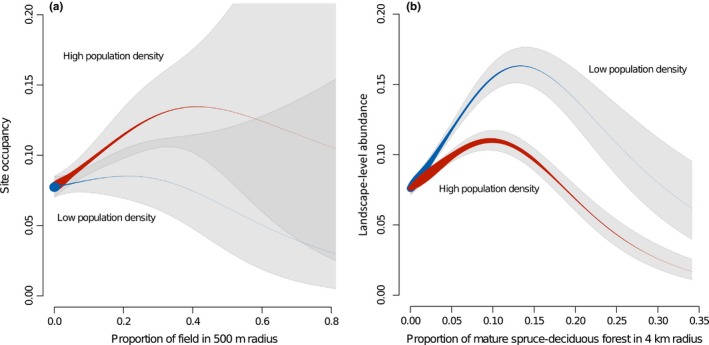
The two strongest interactive effects of regional density on the effect of (a) agricultural fields on site occupancy and (b) preferred habitat (mature spruce and deciduous stands) on landscape‐level abundance of flying squirrel. The partial effects in the best multivariate GAM are presented for the lowest third of population regional density (blue), and the highest third of population density (red) according to the global trend surface model. The line width represents sampling density, and the gray area represents the 95% confidence interval. The darker gray area in part (a) represents overlap of the 95% confidence intervals of high and low population density models. See the model details in the Table 3

Based on two‐dimensional trend surface analyses, the regional density of flying squirrels was higher in western Finland and decreased toward the east and north (Fig. 3). The regional density, that is the global trend surface value, formed significant interactions with proportion of fields, average soil fertility index, and stock volume of grey alder in the site occupancy model (Table 3a) and with all variables except the proportion of forest cover in the landscape‐level abundance model (Table 3b). That is, the positive effect of agricultural fields on site occupancy appeared strong in the high‐density region (western Finland, up to 35% of landscape composition), but was absent in the low‐density region (eastern and northern Finland; Fig. 5a). The site occupancy optimum occurred in slightly more fertile sites in the low‐density region (around 2.7) compared with the high‐density region (around 3.0). The effect of grey alder stock volume on site occupancy was always linear, but was strongly positive in the high‐density region and slightly negative in the low‐density region. However, this pattern was driven by a very small proportion of samples, as alder stock volume remained very low in the great majority of sites.

In the landscape‐level abundance model, the effect of preferred habitat formed by far the strongest interaction with regional density (see the β‐values in the Table 3b). The preference for preferred habitat was significantly higher in the low‐density than in the high‐density region (Fig. 5b). Despite this, there was only small difference in the optimum value for the preferred habitat: around 10% of landscape composition in high‐density area and around 13% in the low‐density area (Fig. 5b). For other variables, despite the significant effects of interactions with regional density were accompanied by low‐effect sizes.

## 4 DISCUSSION

In this study, we distinguish between scale‐dependent effects of habitat characteristics on site occupancy and landscape‐level abundance of flying squirrels, as well as explained geographic differences in the landscape effects. We are unaware of similar attempts to explain the partial components of presence of a species using landscape metrics. The main results of our study are as follows: Landscape variables predicted the placement of population patches (landscape‐level model) at least twice as well as they predicted the occupancy of particular sites (site occupancy model); the clear optimal value of preferred habitat cover for species landscape‐level abundance is a surprisingly low value (10% within a 4 km buffer); landscape metrics exert different effects on species occupancy and abundance in high versus low population density regions of our study area.

### 4.1 Site and landscape components of species presence

Our models clearly predicted placement of population patches better than occupancy of particular sites, that is, the model for landscape‐level abundance produced a considerably better fit than that considering site occupancy. At the site level, extinction‐recolonization dynamics of territories (sites) generate noise in the data because even high‐quality sites can be temporarily unoccupied. The spatial scale that the landscape variables operated on, differed between site and landscape models in the manner we predicted (see also Fletcher et al., 2016; Jackson & Fahrig, 2015; Michael et al., 2016), probably because population‐level processes should affect landscape‐level abundance more than site occupancy. Landscape‐level abundance was predicted well by the landscape variables measured at large spatial scales, in some cases at scales larger than we could measure in this study. Landscape‐scale features are similarly more important than stand‐scale variables in explaining species diversity in birds (Mitchell et al., 2001, 2006). While many earlier mammal studies concentrated on species abundance at small spatial scales, for example in relation to habitat patch characteristics (Bowers & Matter, 1997; Mortelliti et al., 2011), our study supports the view that such studies would benefit from considering large‐scale landscape features when aiming to predict the abundance of species (see also DeCesare et al., 2012; Lindman et al., 2015; Wheatley et al., 2005).

Conclusions about species responses to site and landscape characteristics clearly depend on the habitat measures considered (e.g., Schindler et al., 2013). For example, Michael et al. (2016) concluded that local vegetation structure was more important than forest cover in the surrounding landscape in determining occupancy of 14 reptile species. Unfortunately, we were not able to evaluate site‐level variation in the nest cavity or food resource availability for flying squirrel. Earlier studies indicate that both large aspens providing cavities and alder trees providing food resources are important determinants of occupancy and fitness of flying squirrels (Selonen & Hanski, 2012; Selonen et al., 2016). However, aspen stock volume did not influence flying squirrel site occupancy in the current study, and the effect of alder stock volume was also small. This was an unexpected result, but suggests that the effect of these trees on site occupancy can be detected only at finer scales than we were able to measure in the current study (tree species at 1 km² correlated with flying squirrel presence at 9 ha scale). Instead, both alder and aspen had effects, albeit minor, in the landscape‐level abundance model.

### 4.2 Importance of agricultural fields and movement ability of flying squirrels

Unsurprisingly, the previously observed positive relationship between agricultural areas and flying squirrel occupancy (Santangeli et al., 2013b) weakened (high population density region) or disappeared (low population density region) when soil fertility was accounted for in this analysis. It is clear that fields as such are not a required resource for an arboreal animal like the flying squirrel, although the extent of agricultural land is correlated with soil fertility and the presence of edge habitat (Santangeli et al., 2013b). Nevertheless, it is interesting that even after accounting for soil fertility, fields appeared to have a positive effect on site occupancy in the high‐density region (see discussion below). Furthermore, the landscape‐level abundance of flying squirrels did not decline even if there were 30%–80% of fields in the landscape. This supports earlier studies indicating that the occupancy patterns and movement ability of flying squirrels may not be limited by a lack of structural connectedness even in highly fragmented landscapes (Selonen & Hanski, 2004; Selonen et al., 2012). Nevertheless, the population dynamics of the species are highly dependent on migration patterns (Brommer et al., 2017), which may also be related to the observed patchy density pattern that is characteristic to metapopulations (Hurme et al., 2008).

### 4.3 Habitat threshold

In his review of bird and mammal occupancy patterns, Andrén (1994) concluded that the threshold where occupancy of a species starts to decline sharply lies somewhere between 10% and 30% landscape composition of the habitat (see also Swift & Hannon, 2010). For flying squirrel, landscape‐level abundance declined sharply when the proportion of preferred habitat was below 10%–15% within a 4 km buffer (see also Reunanen et al., 2004). However, the abundance of flying squirrel also declined when the proportion of preferred habitat increased above the optimal level. One explanation for this unexpected pattern could be flexible habitat use and a preference for edge habitats (see below), but also good dispersal capacity that allows the species to inhabit fragmented landscapes (Brommer et al., 2017; Selonen & Hanski, 2004, 2012). Critical habitat thresholds are likely to be species‐specific and our understanding of the mechanism underlying them remains poor, especially for mammals at landscape scales, as studies have concentrated on other species groups such as birds (Swift & Hannon, 2010; but see e.g., Pardini et al., 2010). Thresholds clearly depend on study scale (Swift & Hannon, 2010), and in our case, the site occupancy peaked when the proportion of preferred habitat (250 m buffer) was around 40%.

### 4.4 Habitat use in low versus high‐density regions

We found that landscape variables had different effects in high‐ versus low‐density regions of our study area. For landscape‐level abundance, preferred habitat had higher explanatory power in the low than in the high‐density region in western Finland. This was a surprising result and indicates that further study on flying squirrel habitat preferences in different regions may be needed. In the west (the high‐density region), the species preferred sites near fields and with more grey alder, but this was not the case in the low‐density region. Instead, soil fertility had a higher optimum, and landscapes with black alder were more preferred in the low compared with high‐density region. Some of these differences were very modest, but they nonetheless indicate differences in habitat use between regions. For example, clay soil type is more common in west than in northeast Finland (edge habitat between fields and forest in clay soil is suitable habitat for grey alder), whereas shoreland forests near lakes (suitable habitat for black alder) may be more typical habitat for flying squirrels in particular areas in eastern Finland. Alternatively, our results could indicate density‐dependent habitat use, such that some individuals are forced to use low‐quality habitat when population density is high (Gill et al., 2001; Rozenzweig, 1991; Sundell et al., 2012). However, this hypothesis is not supported by the fact that the response to preferred habitat was not density dependent in the site occupancy model.

In this study, we demonstrated that landscape variables have different effects when occupancy patterns are described at different population levels (site occupancy vs. landscape‐level abundance). One factor that we did not control for was the effect of predators on site occupancy. However, earlier analysis of these (F. G. Blanchet et al. unpublished) and other data (Selonen et al., 2010) indicate that predators do not play a major role in explaining flying squirrel density in the landscape, although it is clear that predators affect individuals locally. We are also unaware of any diseases or parasites that might affect flying squirrel densities. We did not control for historic land use, but the whole study area is under intensive forest management, and the age structure of forests used in the current analysis describes changes in forest structure during the last 50 years. Land use change from forest to agricultural areas has been minor during recent decades in Finland. Thus, in recent history, there have not been major changes in agricultural land area, although the total cropland area has slightly increased in the country (Greenhouse Gas Emission, 1990). Land use in the form of forest cutting during historic times (about >150 years ago) was very intense in western and southwestern Finland (Gyldén, 1850), and historic records from that time indicate that flying squirrels were more abundant in eastern than in western Finland (Mela, 1882). Interestingly, the current situation is the opposite, that is, population density is highest in western Finland, indicating no negative effects from historic forest use on current flying squirrel density.

### 4.5 Conservation implications

A large majority of the study sites occupied in this study are found in landscapes where the proportion of preferred habitat is below the observed optimal value for site occupancy and landscape‐level abundance. This highlights a potential threat to the future survival of the population, in particular in the low‐density region in the northeastern distribution range of flying squirrels in Finland. As the species' population dynamics considerably depend on immigration (Brommer et al., 2017), it is possible that there is a threshold level for the landscape proportion of preferred habitat at which immigration is no longer able to maintain population stability. In this kind of situation, sudden extinctions are possible even over large areas, as has been observed for the Greater glider (*Petauroides volans*) in Australia (Lindenmayer et al., 2011). Based on our results, the optimal landscape for site occupancy by the flying squirrel is a mosaic containing around 35%–40% of mature spruce and deciduous forests situated in semi‐forested landscapes on fertile soils. The optimal size of landscape grain for site occupancy appeared to be in a radius of 250–500 m. This is a larger area than the average female home‐range size (the 100% minimum convex polygon is around 8 ha, i.e., a 160 m radius, Selonen et al., 2001) or current management units used for flying squirrel conservation (radius 10–30 m; Jokinen et al., 2015). The optimal area of preferred habitat within 250 m buffers was around 2 ha, but our analysis cannot be used to evaluate the minimum forest area needed at forest cutting sites containing nest sites of flying squirrels (EU Habitats directive). Instead, we suggest that efficient species conservation follows ecological patterns and should cover, in addition to protection of high‐quality habitat in occupied sites, also its near surroundings, landscape connectivity, protection of temporarily unoccupied sites, and other population‐level effects. For this purpose, conservation might benefit if management considered the scale of functional subpopulations, which based on our results could be an area of at least 1,000 km²—the approximate size of a population patch.

## 5 CONCLUSION

Our result supports the view that large‐scale landscape variables have high power to predict species abundance (Mitchell et al., 2001, 2006). We also conclude that the optimal management plan for species should take into account spatial scales that capture population‐level processes and not only scales that describe the site preferences of individuals. In addition, knowledge of possible regional variation in landscape utilization or density‐dependent habitat use will be essential for the success of a species management or conservation program. This is important even when a species appears to specialize on a certain habitat, as in our case does the flying squirrel, a gliding mammal that requires forest habitat with specific key elements providing food and nesting cavities. We suggest that inclusion of landscape‐level population patterns can significantly increase the precision of biodiversity mapping and prediction of future population trends.

## CONFLICT OF INTEREST

None declared.

## DATA ACCESSIBILITY

The dataset on flying squirrel breeding performance supporting the results of this article is available in the Eurasian Chronicle of Nature (formerly European Boreal Forest Biodiversity, 486 EBFB) database repository, www.earthcape.com. Habitat data are available from the Natural Resources Finland (Luke), www.paikkatietoikkuna.fi.

## AUTHOR CONTRIBUTION

JR and VS conceived the idea, IKH planned and led the flying squirrel inventories, JR and ST prepared the data of landscape metrics, JR performed the data analysis, JR and VS wrote the draft, and all authors contributed comments for the manuscript.
