# Supplementary material for: Multilevel landscape utilization of the Siberian flying squirrel: Scale effects on species habitat use
Source: Ecol Evol. 2017 Sep 7;7(20):8303–15. doi: 10.1002/ece3.3359 (PMC5648651; doi:10.1002/ece3.3359)
Supplement: Supplementary file 1 [file ECE3-7-8303-s001.pdf]

Multilevel landscape utilization of the Siberian flying squirrel: Scale effects on species habitat use

Jaanus Remm, Ilpo K. Hanski, Sakari Tuominen, Vesa Selonen

SUPPORTING INFORMATION

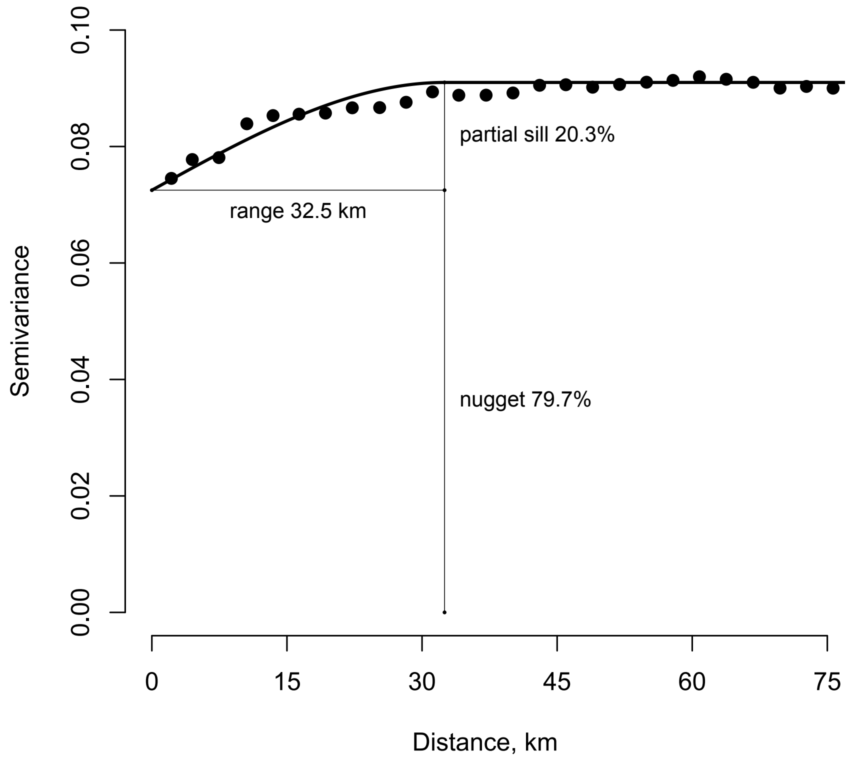

Figure S1. Variogram model representing the spatial autocorrelation structure of flying squirrel site occupancy in Finland.

Table S1. Intercorrelations of explanatory variables. Spearman's rank correlation coefficients (ρ).

**Variable**		**1**	**2**	**3**	**4**	**5**	**6**	**7**	**8**	**9**	**10**	**11**	**12**	**13**	**14**
	**Average soil fertility index**														
1	r = 100 m														
2	r = 250 m	.93													
3	r = 500 m	.83	.94												
4	r = 1 km	.78	.88	.96											
5	r = 2 km	.75	.84	.91	.97										
6	r = 4 km	.73	.82	.88	.93	.97									
	**Proportion of agricultural areas**														
7	r = 100 m	-.10	-.10	-.10	-.09	-.08	-.07								
8	r = 250 m	-.20	-.24	-.27	-.26	-.24	-.20	.29							
9	r = 500 m	-.27	-.33	-.39	-.39	-.36	-.32	.18	.76						
10	r = 1 km	-.26	-.32	-.39	-.41	-.39	-.34	.13	.62	.88					
11	r = 2 km	-.23	-.28	-.34	-.37	-.38	-.34	.10	.52	.74	.90				
12	r = 4 km	-.19	-.23	-.28	-.31	-.32	-.31	.07	.43	.62	.76	.91			
13	r = 10 km	-.11	-.14	-.18	-.19	-.19	-.19	.04	.33	.47	.60	.73	.86		
14	r = 20 km	-.08	-.10	-.13	-.14	-.14	-.13	.02	.30	.42	.53	.66	.78	.94	
	**Proportion of forest cover**														
	r = 100 m	.02	.02	.00	-.01	-.01	-.01	-.14	-.05	-.02	.00	.01	.02	.03	.03
	r = 250 m	.10	.12	.12	.12	.10	.08	-.17	-.35	-.27	-.21	-.17	-.13	-.08	-.06
	r = 500 m	.23	.28	.32	.32	.30	.27	-.14	-.53	-.61	-.52	-.42	-.33	-.23	-.19
	r = 1 km	.27	.32	.38	.41	.39	.36	-.11	-.47	-.61	-.64	-.55	-.44	-.32	-.27
	r = 2 km	.28	.34	.40	.44	.45	.42	-.09	-.41	-.56	-.62	-.63	-.53	-.40	-.33
	r = 4 km	.30	.36	.42	.46	.48	.48	-.07	-.34	-.48	-.56	-.60	-.59	-.46	-.39
	r = 10 km	.22	.25	.3	.33	.35	.35	-.04	-.25	-.36	-.44	-.50	-.54	-.56	-.48
	r = 20 km	.20	.23	.27	.29	.31	.31	-.04	-.22	-.32	-.39	-.44	-.49	-.53	-.50
	**Proportion of mature spruce and deciduous forests, *aka* preferred habitat**														
	r = 100 m	-.43	-.47	-.49	-.50	-.51	-.51	.01	.15	.23	.24	.24	.24	.20	.20
	r = 250 m	-.52	-.57	-.60	-.61	-.62	-.63	.01	.16	.26	.29	.29	.28	.23	.22
	r = 500 m	-.55	-.61	-.65	-.67	-.68	-.69	.02	.12	.22	.26	.27	.27	.21	.21
	r = 1 km	-.57	-.62	-.67	-.70	-.71	-.72	.03	.12	.20	.23	.24	.25	.20	.20
	r = 2 km	-.58	-.63	-.68	-.71	-.73	-.75	.04	.13	.21	.23	.23	.24	.18	.19
	r = 4 km	-.58	-.64	-.68	-.71	-.74	-.77	.04	.15	.22	.24	.24	.23	.18	.18
	**Stock volume within 1 km²**														
	Aspen	-.33	-.36	-.40	-.42	-.42	-.42	.05	.21	.29	.31	.32	.32	.28	.26
	Grey alder	-.39	-.44	-.46	-.48	-.49	-.49	.03	.10	.15	.15	.13	0.1	.02	.01
	Black alder	-.10	-.11	-.14	-.15	-.16	-.17	.03	.08	.08	.09	.08	.08	.07	.07

The table continues on the next page.

Table S1. Continued.

	Variable	15	16	17	18	19	20	21	22	23	24	25	26	27	28	29	30
	**Proportion of forest cover**																
15	r = 100 m																
16	r = 250 m	.70															
17	r = 500 m	.29	.64														
18	r = 1 km	.12	.38	.82													
19	r = 2 km	.05	.26	.64	.87												
20	r = 4 km	.01	.17	.49	.69	.87											
21	r = 10 km	.00	.11	.32	.47	.60	.74										
22	r = 20 km	.00	.09	.27	.39	.48	.59	.84									
	**Proportion of mature spruce and deciduous forests, *aka* preferred habitat**																
23	r = 100 m	.32	.18	-.07	-.17	-.22	-.26	-.21	-.22								
24	r = 250 m	.22	.17	-.09	-.21	-.27	-.31	-.24	-.25	.85							
25	r = 500 m	.14	.11	-.05	-.17	-.24	-.3	-.24	-.26	.75	.92						
26	r = 1 km	.08	.05	-.06	-.12	-.18	-.26	-.21	-.24	.67	.82	.94					
27	r = 2 km	.04	-.01	-.10	-.14	-.16	-.23	-.19	-.23	.62	.77	.88	.96				
28	r = 4 km	.03	-.03	-.14	-.18	-.19	-.21	-.17	-.22	.61	.75	.84	.92	.97			
	**Stock volume within 1 km²**																
29	Aspen	.01	-.09	-.22	-.28	-.31	-.33	-.29	-.27	.27	.33	.35	.36	.37	.38		
30	Grey alder	-.01	-.06	-.15	-.19	-.20	-.22	-.15	-.12	.25	.31	.34	.36	.37	.38	.19	
	Black alder	.01	-.01	-.08	-.13	-.17	-.21	-.18	-.19	.14	.16	.18	.17	.17	.17	.15	.06
